# Attitudes of Heart Failure Patients and Health care Providers towards Mobile Phone-Based Remote Monitoring

**DOI:** 10.2196/jmir.1627

**Published:** 2010-11-29

**Authors:** Emily Seto, Kevin J Leonard, Caterina Masino, Joseph A Cafazzo, Jan Barnsley, Heather J Ross

**Affiliations:** ^5^Divisions of Cardiology and TransplantUniversity Health NetworkToronto, ONCanada; ^4^Faculty of MedicineUniversity of TorontoToronto, ONCanada; ^3^Institute of Biomaterials and Biomedical EngineeringUniversity of TorontoToronto, ONCanada; ^2^Department of Health Policy, Management and EvaluationUniversity of TorontoToronto, ONCanada; ^1^Centre for Global eHealth InnovationUniversity Health NetworkToronto, ONCanada

**Keywords:** cellular phone, heart failure, telemedicine, patient monitoring, attitude

## Abstract

**Background:**

Mobile phone-based remote patient monitoring systems have been proposed for heart failure management because they are relatively inexpensive and enable patients to be monitored anywhere. However, little is known about whether patients and their health care providers are willing and able to use this technology.

**Objective:**

The objective of our study was to assess the attitudes of heart failure patients and their health care providers from a heart function clinic in a large urban teaching hospital toward the use of mobile phone-based remote monitoring.

**Methods:**

A questionnaire regarding attitudes toward home monitoring and technology was administered to 100 heart failure patients (94/100 returned a completed questionnaire). Semi-structured interviews were also conducted with 20 heart failure patients and 16 clinicians to determine the perceived benefits and barriers to using mobile phone-based remote monitoring, as well as their willingness and ability to use the technology.

**Results:**

The survey results indicated that the patients were very comfortable using mobile phones (mean rating 4.5, SD 0.6, on a five-point Likert scale), even more so than with using computers (mean 4.1, SD 1.1). The difference in comfort level between mobile phones and computers was statistically significant (*P*< .001). Patients were also confident in using mobile phones to view health information (mean 4.4, SD 0.9). Patients and clinicians were willing to use the system as long as several conditions were met, including providing a system that was easy to use with clear tangible benefits, maintaining good patient-provider communication, and not increasing clinical workload. Clinicians cited several barriers to implementation of such a system, including lack of remuneration for telephone interactions with patients and medicolegal implications.

**Conclusions:**

Patients and clinicians want to use mobile phone-based remote monitoring and believe that they would be able to use the technology. However, they have several reservations, such as potential increased clinical workload, medicolegal issues, and difficulty of use for some patients due to lack of visual acuity or manual dexterity.

## Introduction

Effective tools to help manage chronic conditions such as heart failure are required if limited health care resources are expected to meet the growing demand [1-5]. Recent studies have found that remote monitoring may be an effective strategy for improving heart failure health outcomes and reducing costs by providing real-time physiological information to health care providers and increasing self-care [6-14]. Mobile phone-based remote monitoring systems are being proposed because mobile phones have considerable computational power while being relatively inexpensive compared to dedicated remote monitoring hardware [15-17]. These systems also have the added benefit of being portable, enabling patients to be monitored anywhere that has mobile phone reception.

Prior to implementing mobile phone-based remote monitoring systems for heart failure management, the willingness and readiness of heart failure patients and their health care providers to use this technology should be determined. A few studies have investigated the perceptions of different patient populations regarding mobile phone-based remote monitoring, such as for asthmatic and hypertensive patients [18-20]. However, heart failure remote monitoring has additional challenges. Heart failure management requires several different parameters to be monitored, resulting in greater complexity, and a delayed response to a worsening heart failure condition could have critical consequences. Furthermore, the average heart failure patient is often older than patients with other chronic illnesses, which could result in them being less willing and able to use certain technologies.

The objective of this mixed methods study was to assess the attitudes of heart failure patients and their health care providers from a heart function clinic in a large urban teaching hospital toward the use of mobile phone-based remote monitoring.

## Methods

### Participants and Recruitment

Study participants (patients and clinicians) were recruited from the Heart Function Clinic at the University Health Network, Toronto, Canada. Eligible patient participants for both the interviews and questionnaires were outpatients diagnosed with heart failure. Other eligibility criteria included being older than 18 years, being able to speak and read in English, not being on the heart transplantation list, and being expected to survive more than 1 year. During their usual heart function clinic visit, all patients who met the inclusion criteria were asked by their cardiologist if they were willing to speak to the study coordinator regarding participating in the study. All patients who were approached for the interviews agreed to participate, and about 12 out of 112 patients approached to participate in the survey declined (11%). See Table 1 for demographic/clinical characteristics of the patient participants.

Clinician participants were physicians and nurse practitioners associated with the Heart Function Clinic. Clinicians were sent an email asking them to respond if they would like to participate in the study. All clinicians who were emailed agreed to be interviewed. The clinicians included 5 staff cardiologists, 5 nurse practitioners, and 6 clinical fellows.

### Study Setting and Design

We asked 100 heart failure patients to complete and mail back a questionnaire that included questions on their perceptions of remote monitoring and their comfort with using mobile phones and computers. The questionnaires were administered between September 2009 and February 2010. The participants were asked to rate each of the 8 questions using a five-point Likert scale (Table 2). The estimated time to complete the questions was approximately 5 minutes. If patients did not return the questionnaire within 2 weeks, they were called to remind them to do so. Those who still did not return the questionnaire were called once again after another 2 weeks. The final response rate was 94 out of 100 administered questionnaires (94%).

Individual face-to-face semistructured interviews were also conducted with 20 heart failure patients (different patients from those surveyed) and 16 heart failure clinicians to elicit their attitudes toward mobile phone-based remote patient monitoring. Informal caregivers (eg, parents or children of the patients) were also present at the patient interviews, approximately a fifth of the time, and were encouraged to offer their opinions. The interviews were conducted between April 2008 and February 2009. The interviews were recorded and later transcribed. Each interview lasted between 30 and 60 minutes. The transcripts were analyzed using a conventional content analysis approach [21]. Two researchers (ES and CM) analyzed the transcripts independently and coded the transcripts with the software program NVivo version 7 (QSR International, Doncaster, Victoria, Australia). The researchers then discussed the themes and issues that emerged until a consensus was reached.

The study was approved by the University Health Network and University of Toronto Research Ethics Boards.

### Description of a Mobile Phone-Based Remote Monitoring System

A description of a mobile phone-based remote monitoring system was provided to all study participants prior to eliciting any feedback. Patients were also walked through a prototype system, demonstrating the steps that they would have to take for the proposed remote monitoring. (See Multimedia Appendix 1 for the description and instructions for using the proposed monitoring system.) The described system included a wireless (Bluetooth-enabled) weight scale, blood pressure monitor, and single-lead electrocardiogram (ECG) recorder that automatically transmitted the data to a mobile phone. Patients were expected to take their weight and blood pressure (pulse would be included with the blood pressure measurement) every morning and an ECG recording weekly. They were also asked to record their symptoms each morning by answering symptom questions by pressing 1 for no and 2 for yes on the mobile phone keypad. The mobile phone automatically transferred the data to computer servers for analysis using third-generation (3G) technology. Depending on the readings, an alert message could be generated and sent to the patient’s mobile phone. When an alert was generated, an email was also sent to a cardiologist’s mobile phone with all relevant patient information. Both patients and clinicians were able to view all historical data and alerts on a secure password-protected website.

## Results

### Survey Results

Table 1 summarizes the demographic and clinical characteristics of the patients who completed and returned the questionnaire. The demographics of the participants are representative of the patient population who attend the University Health Network Heart Function Clinic.

Table 2 summarizes the results from the survey. The patients indicated that they thought it was important to monitor their weight and blood pressure. They were slightly more comfortable using a mobile phone than a computer (*P* < .001, 2-tailed paired Student *t* test, *t*_89_ = 4.13), but rated the comfort level high for both. Most patients could easily access a computer. Patients rated their confidence in looking up health information on a mobile phone and computer equally high. Patients indicated moderately high confidence that their privacy would be secure if their health information was accessible by a computer.

**Table 1:** Demographic and clinical characteristics of patient participants who returned a completed survey (missing values account for totals less than 94 )

Variable		Response, N=94
Mean age, years (SD)		54.6 (13.4)
Gender	Male	74 (79%)
	Female	20 (21%)
Ethnicity	Caucasian	71 (76%)
	African Canadian	7 (8%)
	Southeast Asian	4 (4%)
	Chinese	4 (4%)
	Other	7 (8%)
Marital status	Married	62 (67 %)
	Never married	17 (18%)
	Divorced	10 (11%)
	Widowed	4 (4%)
Highest education achieved	Less than high school	7 (8%)
	High school	25 (27%)
	Trade or technical training	16 (17%)
	College/university undergraduate	37 (40 %)
	Postgraduate	8 (9%)
Income	< $15,000	20 (21%)
	$15,000 - $29,999	17 (18%)
	$30,000 - $49,999	17 (18%)
	$50,000 - $74,999	14 (15%)
	> $75,000	14 (15%)
	Preferred not to answer	12 (13%)
Employment	Full-time	27 (29%)
	Part-time	4 (4%)
	Disabled	37 (40 %)
	Retired	15 (16%)
	Unemployed	11 (12%)
New York Heart Association class	II	40 (43%)
	II/III	12 (13%)
	III	38 (40%)
	IV	4 (4%)
Mean left ventricular ejection fraction (SD)		26.8 (8.6)
Mean length of heart failure, years (SD)		6.3 (6.7)
Primary cause of heart failure	Ischemic	32 (34%)
	Idiopathic	47 (50%)
	Other	15 (16%)

**Table 2:** Mean responses to survey questions (1: Strongly Disagree, 2: Disagree, 3: Neither Agree or Disagree, 4: Agree, 5: Strongly Agree)

Survey Question	Mean Response (SD)
I need to weigh myself every day at home.	4.5 (0.8)
It is important to take my blood pressure at home as often as my doctor says I should.	4.3 (0.9)
I am confident that my privacy would be secure if my health information was accessible by a computer.	3.9 (1.2)
I feel comfortable using a mobile phone.	4.5 (0.6)
I feel confident that I could use a mobile phone to look up my health information if shown how to do it.	4.4 (0.9)
I feel comfortable using a computer.	4.1 (1.1)
I feel confident that I could use a computer to look up my health information if shown how to do it.	4.4 (0.9)
It is easy for me to get access to a computer at home.	4.4 (1.1)

### Interview Results

Heart failure patients and their health care providers perceived numerous benefits and barriers to using mobile phone-based remote monitoring. Table 3 summarizes the benefits and Table 4, the barriers. The willingness and readiness of the patients and clinicians to use a mobile phone-based remote monitoring system are presented separately below, and are partially informed by the perceived benefits and barriers.

#### Willingness to Use Mobile Phone-Based Remote Monitoring

Most patients perceived that monitoring their weight and blood pressure was important to help manage their heart failure condition. Several interviewed patients volunteered without prompting to use the monitoring system whenever it was made available. Interviewed patients stated that they would be willing to try using the proposed remote monitoring system under the following conditions and caveats:

First, the monitoring system should be an adjunct to their relationship with their clinician at the heart function clinic. It should not be a replacement.

Second, patients would adhere to taking daily measurements long-term if they perceived clear tangible benefits from using it. The patients also stated that they would monitor their weight, blood pressure, and other factors more closely if their heart condition ever worsened.

Third, the system should be as easy to use as possible. They also requested appropriate training and a way to get technical support if they needed it.

Fourth, some patients questioned the necessity of monitoring their blood pressure daily. Some patients did not believe that they needed to take their blood pressure daily because their blood pressure in the past had been stable.

The clinicians thought that the proposed remote monitoring system could help them manage their patients’ condition by providing timely alerts to worsening health and additional information about their patients that they would otherwise not have. They also believed that the monitoring system could improve their patients’ self-care. All interviewed clinicians were willing to try using the monitoring system under the following conditions and caveats:

First, the system should not result in a significant increase in workload for them. The clinicians stated that they did not have further capacity to take on duties that would add to their already busy schedule. In particular, they were concerned about managing the alerts during off-hours (during nights, week-ends, and vacation). The clinicians suggested that a nurse practitioner be assigned to initially respond to the alerts and to contact the cardiologists as necessary.

The medicolegal implications of using the monitoring system needed to be determined. Clinicians were concerned that they would be legally liable if they did not respond to an alert immediately and the patient’s health worsened as a result. Clinicians recommended that a method was necessary to document their actions from the alerts for medicolegal purposes.

The patient alerts and instructions needed to be appropriate and safe. Clinicians were concerned that the alerting algorithm would generate inappropriate alerts and instructions to the patient. Some suggested that a health care provider should vet each alert before it was sent to the patient.

#### Ability to Use Mobile Phone-Based Remote Monitoring

Patients generally thought that they would be able to use the proposed monitoring system. Many of them already practiced some form of self-monitoring, including weighing themselves in the morning and taking their blood pressure periodically with their own home blood pressure monitor. All patients who owned home blood pressure monitors and weight scales thought they were easy to use. In addition, some patients had access to a computer and many already owned a mobile phone. Several of the patients who were not accustomed to the technology stated that they would be able to receive help from family members (eg, their spouses and children).

Both the interviewed patients and clinicians thought that older and less technologically savvy patients could have trouble operating the mobile phone. In particular, they thought that the small buttons and font on the mobile phone could cause difficulty to some patients. However, none of the interviewed patients thought that they themselves would have significant problems using the equipment.

The interviewed clinicians did not have concerns on their ability to use the system but instead cited barriers related to the readiness of the clinic and the health care system to support the use of remote monitoring. For example, additional human resources would be required at the clinic, such as a nurse practitioner, to respond to the alerts at all times. Another concern was that there was no method of remuneration for phone interactions with their patients.

**Table 3:** Perceived benefits by patients and clinicians (quotes in italics)

Benefit	From Patient Interviews	From Clinician Interviews
Clinical care improvement	Clinicians would be able to view their patients’ health data easily and quickly. The alerts sent to the physicians would enable them to provide their patients with immediate feedback.	Clinicians would be able to monitor their patients closely and would be provided with more information than they previously had to base their clinical decisions on. The information would be particularly useful for medication titration, and could help with false high blood pressure seen in clinic (ie, white coat syndrome). The alerts would be beneficial to inform them when their patients needed their help the most.
	*The fact that it goes to a hospital and to a team of professionals that could give me feedback about where I am in my health and to be able to direct me to stay on track and that all this technology is grouped together in order to help me that way. I think that’s star quality treatment.*	
Self-care improvement	The system would improve the patient’s understanding of how lifestyle choices would affect their health and would help them keep track of their health (“body awareness”). The system would also help them get into a routine and inform them when they are not at their ideal target range for their weight and blood pressure.	Clinicians thought the system would help reinforce the instructions that were given to their patients in clinic (eg, following reduced salt and fluid intake).
	*It gives you a vision of how things are going...it's probably easier for you to make slight adjustments also to your eating habits and that will allow you to better treat your health, better treat your symptoms.*	*We throw a lot of information at them and they probably don't get half of it and they can come home and this is a bit of a security blanket.*
Increased reassurance/ accountability	Patients and their caregivers would feel reassured that their doctors would be watching over them. They also thought they would feel a sense of accountability because they would be closely watched, which would have a positive effect of keeping them adherent to their self-care regimen, including diet and exercise.	Not mentioned in the interviews.
	*You learn about your foods and your exercise, smoking, drinking and all that stuff, but this would kind of give you motivation to stay within say a weight range all the time and it's almost like a trainer.*	
Reduced clinic visits	The number of times they would have to visit the clinic would be reduced. Many patients stated that they traveled far distances to get to their scheduled clinic visits, which was inconvenient for themselves and their family members.	Clinic visits by some patients could be reduced if they were closely monitored at home.
Ability to monitor even if they were away from home	Patients would be able to bring it with them on vacation (eg, Florida) and to their cottage.	Not mentioned in the interviews.
	*It's not ready of course but I'm leaving for Florida in a couple of days or so, well, for the month of March. I could take it with me if I was on the system.*	

**Table 4:** Perceived barriers by patients and clinicians (quotes in italics)

Barrier	From Patient Interviews	From Clinician Interviews
System not suitable for all patients	Patients with poor vision could have trouble reading the mobile phone screen, and patients with inadequate manual dexterity could have problems entering information on the mobile phone keypad. However, none of the interviewed patients thought they themselves would have these problems. Patients also had concerns of getting used to the technology, but they thought they would be able to learn to use it with technical support and training. Some patients stated that their family members could help them use the technology.	Clinicians echoed the concerns expressed by the patients that some would have difficulty using the proposed monitoring system. In addition, they were concerned that patients predisposed to anxiety might not be suitable to use it.
		*You never want to overload people because not everybody is a real techy kind of person and you're dealing with an older population that's not really inclined. A lot of these patients are going be intimidated at first, you know, and will just need some gentle training but I have no doubt that you can train people to do this because we've trained them to take transplant medications.*
Clinical workflow challenges	Clinicians responding to the alerts could be “overburdened”, especially if time was not specifically allocated for managing the alerts.	Clinicians are too busy to respond to the alerts. They were concerned about managing the alerts 24/7, including when they were away on vacation. The most common suggestion was to have a nurse practitioner respond to the alerts. They also commented that there should be a way to financially reimburse physicians for calling patients.
	*I think they would just get bombarded by calls every time you had a symptom.*	
Medicolegal issues	Not mentioned in the interviews.	There could be legal implications if clinicians did not respond to an alert immediately and the patient’s health further deteriorated. They thought that a method to document their actions would be necessary for medicolegal reasons.
Inappropriate automated instructions	The system might instruct them to go to the emergency department (ED) unnecessarily, which would contribute to the backlog in the ED. They were also concerned about the anxiety that unnecessarily urgent alert messages could cause.	The automatically generated instructions and alerts sent to the patients could be inappropriate. Some clinicians suggested that a clinician should vet each alert before the alert is sent to the patient.
Security/ privacy	In general, patients did not have major security concerns about using the monitoring system as long as reasonable measures were taken to protect the confidentiality of their information.	The patient information must be secure, and appropriate technological measures must be taken to ensure patient confidentiality.

## Discussion

### Willingness to Use Mobile Phone-Based Remote Monitoring

Heart failure patients and their health care providers perceived a large opportunity for remote monitoring to increase self-care and improve clinical care. Patients thought that remote monitoring would provide a sense of reassurance. This feeling of reassurance was also found in a previous trial investigating remote monitoring of patients with implantable cardioverter-defibrillators for cardiac resynchronization therapy [22]. However, patients did not want remote monitoring to result in a decrease in communication with health care providers, and thought that they would continue remote monitoring only if there were clear and tangible benefits to their doing so. These findings were supported by a study investigating the views of patients with type 2 diabetes on self-monitoring of blood glucose [23]. It found that self-monitoring decreased over time largely because patients did not know how to interpret and act on the blood glucose readings and they perceived a lack of interest by their clinicians in their readings.

Our findings had similarities to the results from studies examining the attitudes of patients and health care providers on using mobile phone-based remote monitoring with other patient populations. A study of the acceptability of mobile phone-based remote monitoring of hypertensive patients found that the patients and clinicians were willing to try using the technology because they perceived that it would encourage self-care through improved medication and lifestyle behavior adherence, and that it would help detect health deterioration earlier than without its use. The study found that clinicians were concerned about the increase in workload and the need to respond immediately to the continuous incoming blood pressure information [18]. Studies with asthma patients also found high levels of acceptability in using mobile phone-based remote monitoring [19, 20]. The perceived benefits included identifying poor control of the asthma condition quickly and reducing the need for face-to-face consultations. Both patients and clinicians cited increased clinical workload and implementation costs as concerns.

### Ability to Use Mobile Phone-Based Remote Monitoring

The survey data indicated that patients were comfortable using mobile phones and computers, and were confident that they could learn to look up health information on both mobile phones and computers. In addition, many patients already use home medical devices, such as weight scales and blood pressure monitors. The perceived readiness of the patients to use mobile phone-based remote monitoring technology is in contrast to the findings of a study with asthma patients [20]. The asthma study had a low survey response rate by the clinicians and patients, and had a high rate of return of uncompleted questionnaires that stated there was a lack of perceived relevance. The researchers concluded that mobile phone-based remote monitoring was not of interest to the majority of the participants, and remained an interest only to early adopters of technology.

It is possible that the increased ubiquity of mobile phones between the study of Pinnock and colleagues in 2005 and our study in 2010 is partly responsible for this difference. Our study participants rated their comfort of using a mobile phone higher than using a computer, a difference that was found to be statistically significant (*P* < .001). A wireless market study report found that, in 2006, mobile phone ownership was much higher for younger Canadians than those 55 years or older, but that the usage among older Canadians had grown more between 1997 and 2006 [24]. A systematic review of studies investigating mobile phone voice and text messaging interventions for health care found improvements in outcomes of care and processes of care, and suggested a “trend toward a digital divide in the reverse” [25].

Undoubtedly, the use of mobile phone-based remote monitoring is not suited for all heart failure patients, as acknowledged by both the interviewed patients and the clinicians. For example, patients with poor manual dexterity or vision and those who are predisposed to high anxiety may not be suitable candidates for the use of this technology. However, all 20 of the interviewed patients thought that they themselves would be able to use the proposed technology. This was similar to the finding in a trial of mobile phone remote monitoring of asthma patients, where the interviewed patients hypothesized that patients less comfortable with mobile phones might have greater difficulty using the equipment, but none of the patients inexperienced with mobile phones actually reported problems [19]. Future investigation is warranted into whether the perception of the percentage of patients who would be unable to use mobile phone-based remote monitoring is higher than in reality.

A factor that could influence the ability of patients to successfully use a mobile phone-based monitoring system is its design. A user-centric design process to develop a simple and easy-to-use system could significantly increase the number of patients who could successfully use the technology. The interviewed patients stressed the importance of developing a system that is robust and as easy to use as possible, and that technical support will be required. Studies have shown that weaknesses in telemedicine implementations are largely attributed to technical problems [19, 26]. Another factor to success is the availability of informal caregivers to help. Many of the patients stated that they had spouses and children who were much more technologically savvy than they were and that these relatives could help the patients use the monitoring system.

### Limitations

Participants in this study were recruited from a single heart function clinic. This particular clinic treats a higher proportion of severely ill patients compared to other heart function clinics. Patients attending this clinic include young heart failure patients (eg, in their 20s). The average age of the heart failure patients attending the clinic is approximately 54 years (SD 15 years), which is consistent with the participants in this study. Therefore, it is possible that the study participants might be slightly more comfortable than the average heart failure patient with using technology. Another limitation is that the patients who agreed to participate in the study may have been biased to have a more positive attitude toward remote monitoring. However, the participation refusal rate was very low, which suggests that the bias was minimal. Finally, the mobile phone-based remote monitoring system that was proposed to the participants had functionality that was beyond what is available in current best practice. A description of the functionality of currently available systems may have elicited less positive  responses.

### Conclusions

The heart failure patients participating in this study were confident in their ability to use a mobile phone-based remote monitoring system, largely because mobile phones are becoming increasingly pervasive even among older individuals. The patients and clinicians were willing to use a mobile phone-based remote monitoring system because they perceived many benefits, including providing patients with immediate feedback at the earliest sign of deteriorating health. However, both groups cited several caveats to their willingness to use such a system. The monitoring system would have to be easy to use, the benefits to using the system must be evident and tangible, patient information must be secure, and any automated instructions or feedback to the patient must be trusted. Reservations by the clinicians regarding using the system included increased clinical workload and medicolegal issues. If the concerns voiced by the patients and clinicians are first addressed, mobile phone-based remote monitoring could be a relatively inexpensive and convenient tool to improve heart failure management.

## Multimedia Appendix

Description provided to study participants of the proposed mobile phone-based heart failure remote monitoring system.
