# Supplementary material for: Attitudes of Heart Failure Patients and Health care Providers towards Mobile Phone-Based Remote Monitoring
Source: J Med Internet Res. 2010 Nov 29;12(4):e55. doi: 10.2196/jmir.1627 (PMC3056531; doi:10.2196/jmir.1627)
Supplement: Supplementary file 1 [file jmir_v12i4e55_app1.pdf]

Multimedia Appendix 1: Description Provided to Study Participants of the Proposed Mobile Phone-based Heart Failure Remote Monitoring System

Prior to eliciting any feedback from the study participants, a description of the proposed mobile phone-based remote monitoring was provided and a walk-through of a prototype system was conducted demonstrating the steps that the patients would have to perform for the remote monitoring. Below are the description provided and the instructions for using the proposed monitoring system.

Monitoring System Description

The heart function home monitoring system consists of special wireless (Bluetooth) home medical devices, a pre-programmed BlackBerry cell phone, and a central data storage system that is located in the hospital.

Weight, blood pressure/pulse, and symptoms are taken at home and then transmitted wirelessly to the BlackBerry. You might also be instructed to take an Electrocardiogram (ECG) at home as well. The BlackBerry then processes the readings and sends the information automatically to the computer at the hospital. The BlackBerry also stores the results and displays graphs of the measurements. You will be provided with instructions on the Blackberry screen once you have completed all the daily measurements. Your healthcare team at the Heart Function Clinic will also be alerted if your measurements are out of the goal range.

Instructions for Home Heart Function Monitoring

You will be asked to take your **weight, blood pressure/pulse, and symptoms first thing in the morning EVERY day**. If you were provided with an ECG recorder, you will be asked to take an ECG reading at least once a week.

Taking your Morning Weight

You will be taking your weight EVERY morning. Please remember to do the following:

- Go to the washroom (urinate) before you take your weight
- Undress before you take your weight
- Take your weight BEFORE you eat or drink anything

To take your weight:

1. Tap the blue START button on the front edge of the weight scale with your foot.
2. After the scale zeros (the scale will display 0.0 lb), step on the scale with both feet.

3. Once your weight is displayed on the scale, step off the scale. The weight measurement will automatically be sent to the BlackBerry.
4. Answer the "Was this?" question on the BlackBerry by pressing 1 for "first thing in the morning".

Note: Only the weight measurements taken first thing in the morning are important to determine your heart health. Weight measurements that are marked as "during the day" will NOT be used to send alerts to you or your healthcare team.

Taking your Blood Pressure/Pulse

You will be taking your blood pressure EVERY morning. Your pulse (heart rate) will be automatically recorded when you take your blood pressure. When you are taking your blood pressure, please remember to do the following to get the most accurate measurement:

- Sit comfortably in a chair with a table in front of you. Make sure both feet are flat on the floor.
- Make sure you relax for about 5-10 minutes before taking the measurement.
- Remain still and keep quiet during the measurement.
- Always take the blood pressure on the same arm.

To take your blood pressure/pulse:

1. Put on the arm cuff by wrapping the cuff around the upper arm, about 2-3 cm above the elbow. Make sure you take your blood pressure always on the SAME arm. Place the cuff directly against the skin, because clothing may cause a faint pulse, resulting in a measurement error. Make sure the arrow on the cuff is pointing down towards your fingers.
2. Adjust the tightness so that two fingers can slide under the top and bottom cuff edge as shown in Figure 1 below.
3. Position the air tube off-center toward the inner side of the arm, in line with the little finger.
4. Rest your arm on the table. Make sure the center of the cuff is at the same height as your heart.
5. Press the START button on the blood pressure monitor. The last blood pressure measurement is displayed briefly. The pressure indicator will be displayed as the cuff inflates and then slowly deflates. It is normal for the cuff to feel very tight.
   Note 1: If you wish to stop inflation at any time, press the START button again.
   Note 2: If an appropriate pressure is not obtained, the monitor will try again by re-inflating the cuff.
6. When the measurement is complete, the systolic (higher) and diastolic (lower) blood pressure readings and pulse rate are displayed on the blood pressure monitor. You may now take off the cuff. The blood pressure and pulse measurements will be sent automatically to the BlackBerry.
7. Answer the "Was this?" question on the BlackBerry by pressing 1 for "first thing in the morning".

Taking your Symptoms

You will be taking your symptoms EVERY morning. To take your symptoms:

1. Go to the Main Menu on the BlackBerry by pressing 1 from the Summary screen.
2. Select "Symptoms" by pressing 2.
3. Select "Record Symptoms" by pressing 2.
4. Answer the displayed questions by pressing the numbered buttons on the BlackBerry. If you answer "yes" to any of the first 5 questions (first 6 questions if you have an implanted cardioverter defibrillator), you will be asked an additional 5 questions.

Note: If you have entered an incorrect answer to a symptom question, you have an option to cancel by pressing 3 on the yes/no symptom questions. You can then start again by going back to step 1 of taking your symptoms.

Recording an ECG

If you were provided with an ECG recorder, you will be recording your ECG (heart rhythm) **at least once a week**. When you are recording your ECG, please remember to do the following to get the most accurate recording:

- Sit comfortably when recording your ECG.
- Remain as still as possible and do not speak when recording your ECG.
- Do not move the ECG device when recording your ECG.
- Make sure the contact between your right hand and the large side electrode (metal plate) is good.
- Make sure the contact between the two bottom electrodes and the skin on your chest is good.

To record your ECG:

1. Go to the Main Menu on the BlackBerry by pressing 1 from the Summary screen.
2. Press 6 on the BlackBerry to get to the ECG Menu.
3. Press 2 on the BlackBerry to select "Record ECG".
4. Press the "ON" button (left button) on the ECG recorder.
5. Hold the ECG recorder in your right hand with the hand electrode (metal plate) in contact with your hand. Press the bottom two electrodes firmly to the skin of the middle of your chest.
6. In a few seconds, the ECG recorder will start making a high-pitched noise for about 30 seconds while your ECG is being recorded. Do NOT move during the 30 seconds. When the noise stops (the BlackBerry will show a complete circle on the screen), you can let go of the ECG recorder.
7. Look at the recording on the BlackBerry. If the recording shows an ECG (that is, you see periodic spikes), then answer "yes" to the question "Save reading?" by pressing 2. If you want to retake the ECG because you lost good contact with the

electrodes during the recording, then press 1 for no, and repeat the instructions to take an ECG.

Taking Measurements during the Day

If you are feeling unwell during the day and would like to use the monitoring system, first take your symptoms as described in the "Taking your Morning Symptoms" section above. You may then be instructed to take your blood pressure/pulse (and ECG).

You may also choose to take your blood pressure/pulse (and/or ECG) some time during the day, other than first thing in the morning. You can take your blood pressure/pulse (and/or ECG) as described above, but after taking the measurement when asked on the BlackBerry "Was this?", answer "During the day" by pressing 2.
